# Supplementary material for: RAN translation at C9orf72-associated repeat expansions is selectively enhanced by the integrated stress response
Source: Nat Commun. 2017 Dec 8;8:2005. doi: 10.1038/s41467-017-02200-0 (PMC5722904; doi:10.1038/s41467-017-02200-0)
Supplement: Supplementary file 1 — Supplementary Information [file 41467_2017_2200_MOESM1_ESM.pdf]

# Supplementary Figure 1

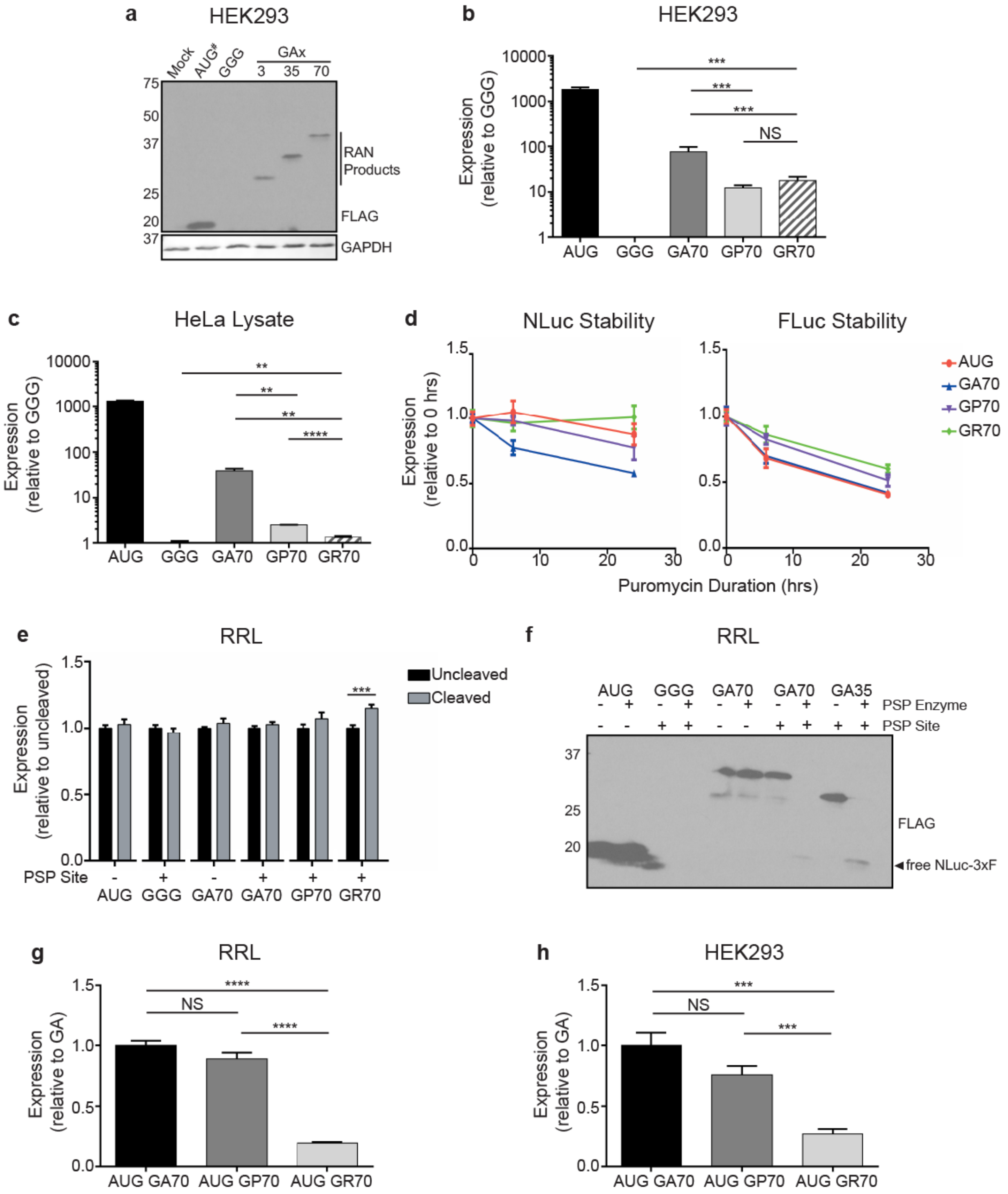

**Supplementary Figure 1: C9RAN translation reporter system allows for quantitative assessment of RAN translation in all three sense reading frames.**

(a) Anti-FLAG western blot analysis of control and GA C9RAN translation reporters in HEK293 cells. #One-tenth AUG reporter was transfected into cells to prevent over-exposure. (b) Expression of control and C9RAN reporters in mRNA transfected HEK293 cells, n=6. (c) Representative expression of control and C9RAN reporter mRNAs in HeLa cell lysate, n=3. (d) The stability of control and C9RAN reporter proteins was assessed in transfected HEK293 cells by treating cells with 10 μg/mL puromycin and measuring reporter activity at 0, 6, and 24 hours later, n=9 (0 and 6 hours), n=12 (24 hours). (e)The hindrance of the DPR fusion on NLuc activity was assessed in RRL by incubating completed reactions with PSP enzyme to cleave DPRs from NLuc, n = 6-9. (f) Anti-FLAG Western blot to confirm PSP cleavage of C9RAN fusion proteins in RRL. (g) Expression from AUG-driven reporters for each sense reading frame, relative to AUG-GA70, in RRL, n=6 and (h) HEK293 cells, n=6. RRL, rabbit reticulocyte lysate. Graphs in (b) and (c) represent mean ± SD. Remaining graphs show mean ± SEM. Two-tailed Student's t test with Welch's correction, $**p < 0.01$; $***p < 0.001$; $****p<0.0001$.

## Supplementary Figure 2

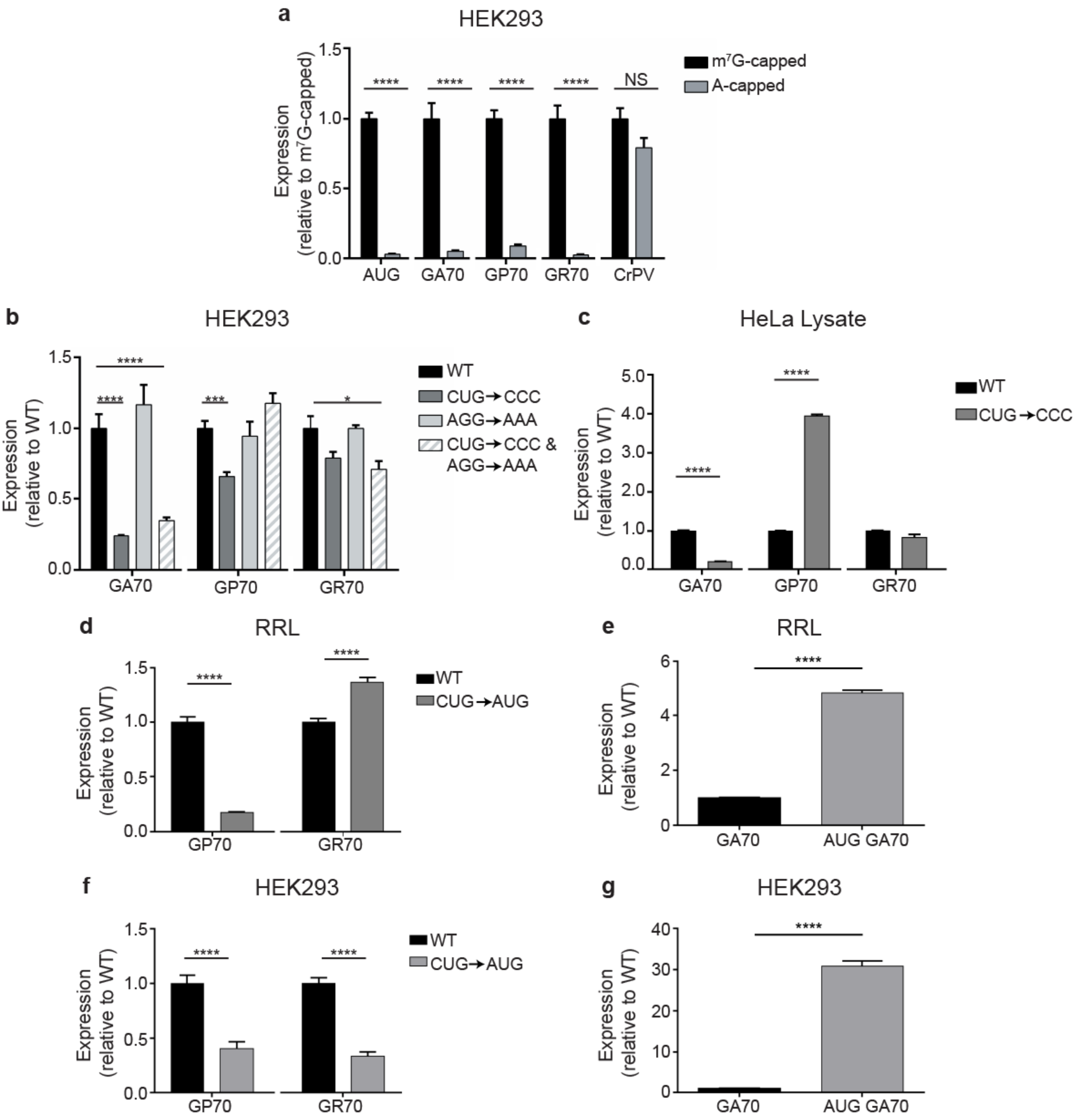

**Supplementary Figure 2: C9RAN translation in HEK293 cells is cap-dependent and can initiate at a near-cognate start codon.**

(a) Expression of $m^7G$-capped and A-capped control and C9RAN mRNA reporters in HEK293 cells, n=9. (b) Expression of all three sense C9RAN mRNAs in HEK293 cells following mutation of near-cognate codons in GA frame, n=6. (c) Expression of sense C9RAN mRNAs in HeLa cell lysate with or without CUG codon mutated to CCC, n=11. (d) Expression of GP and GR-NLuc reporters in RRL from constructs with CUG codon mutated to AUG, relative to WT sequence in RRL, n=6. (e) Insertion of an AUG codon upstream of the repeat in the GA frame enhances GA-NLuc expression in RRL, n=6. (f) Mutating CUG codon to AUG decreases expression of GP and GR-NLuc reporters in HEK293 cells, n=6. (g) Insertion of an AUG codon upstream of the repeat in the GA frame enhances GA-NLuc expression in HEK293 cells, n=24. Graphs represent mean ± SEM. Two-tailed Student’s t test with Welch’s correction, $*p < 0.05$; $***p < 0.001$; $****p<0.0001$.

## Supplementary Figure 3

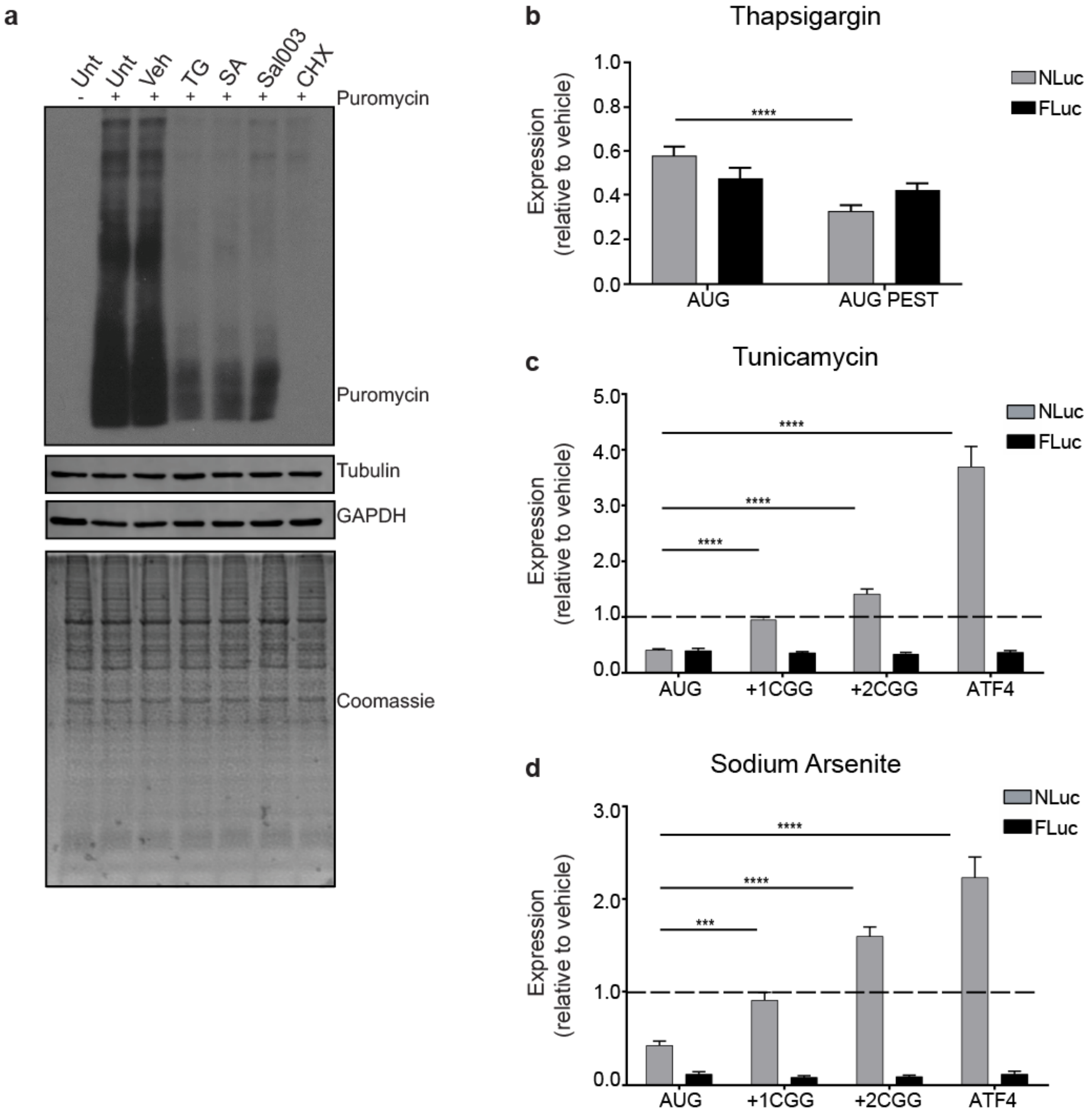

**Supplementary Figure 3: CGG RAN translation in multiple reading frames is refractory to translation attenuation during ER and oxidative stress.**

(a) Anti-puromycin western blot of cells treated with various cell stress inducers and 10 μg/mL puromycin to monitor global translation activity. Tubulin, GAPDH, and Coomassie stain were used as loading controls. Unt, untreated. Veh, vehicle. TG, 2μM thapsigargin. SA, 10μM sodium arsenite. Sal003, 20μM Salubrinol. CHX, 100μg/mL cycloheximide. (b) Destabilization of AUG-NLuc reporter with PEST tag results in greater decrease in AUG-NLuc expression with TG treatment. (c-d) Expression of control and CGG RAN NLuc reporters in transfected HEK293T cells when treated with (c) 2.5 μg/mL Tunicamycin, n=9 and (d) 20 μM sodium arsenite, n=9, for 5 hours. FLuc was co-expressed as an internal control with each NLuc reporter. AUG and ATF4 reporters serve as reporters that are attenuated and stimulated, respectively, during activation of the ISR. Graphs represent mean ± SEM. Two-tailed Student's t test with Welch's correction, $***p < 0.001$; $****p<0.0001$.

## Supplementary Figure 4

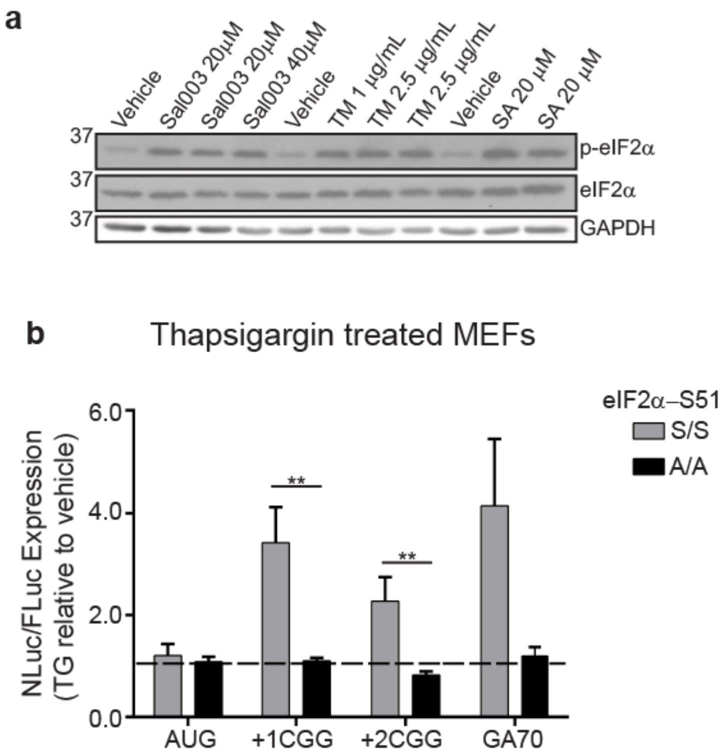


**Supplementary Figure 4: Thapsigargin-induced enhancement of C9 and CGG RAN translation requires phosphorylated eIF2α**

(a) Western blot showing increased phosphorylation of eIF2α in HEK293 cells following treatment with Sal003 (20 or 40 μM), TM (1 or 2.5 μg/mL), and SA (20μM). (b) Expression of control, CGG, and C9RAN NLuc reporters normalized to co-transfected FLuc in WT and eIF2α-S51A/A homozygous mutant MEFs following treatment with 1 μM thapsigargin (TG), n=6-9. Graph represent mean ± SEM. Two-tailed Student's t test with Bonferroni and Welch's correction, $**p < 0.01$.

## Supplementary Figure 5

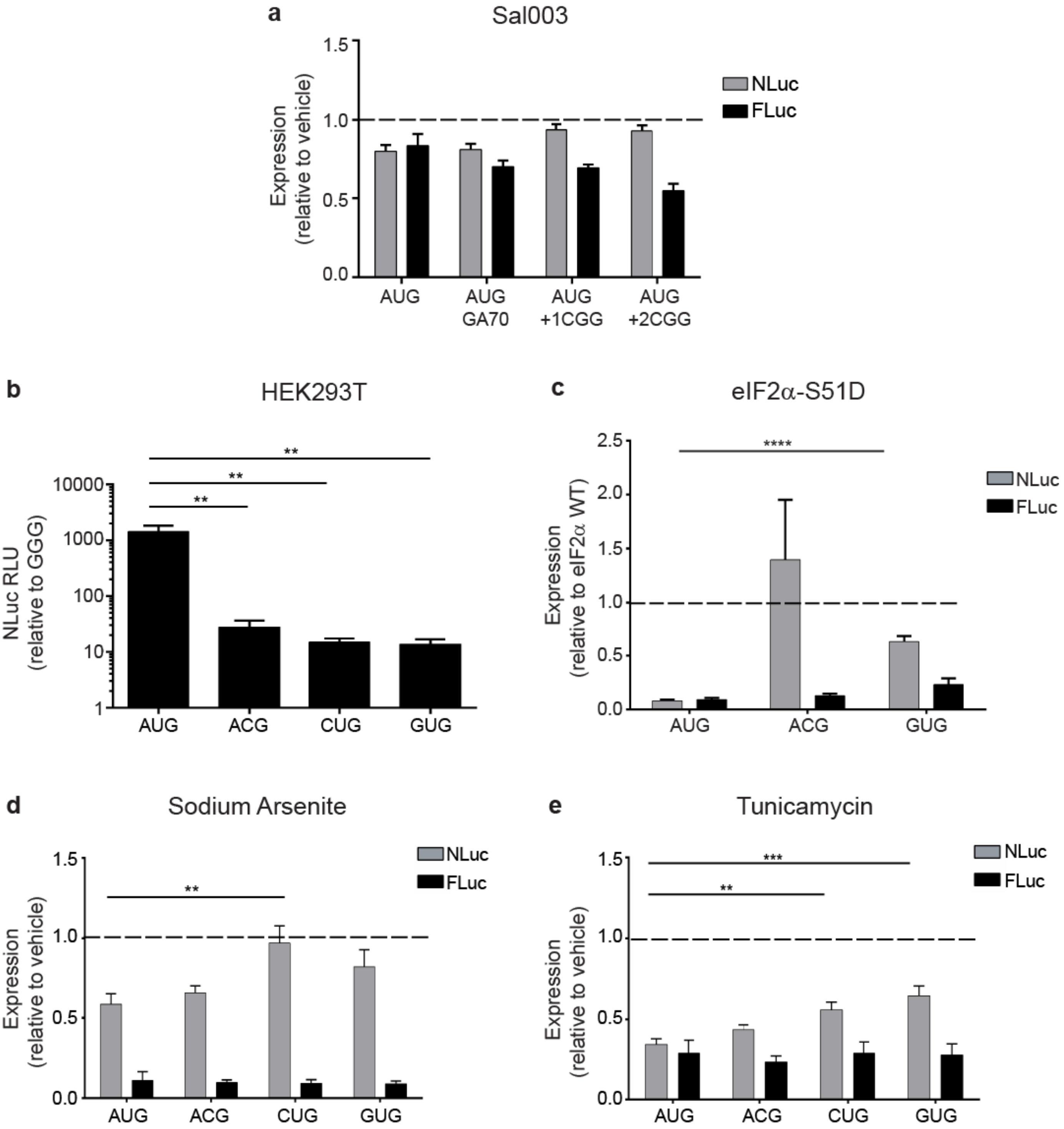

**Supplementary Figure 5: Initiation at near cognate codons is refractory to multiple stress stimuli.** (a) Expression of the control AUG-NLuc and AUG-initiated reporters harboring 100 CGG or 70 $G_4C_2$ repeats in multiple reading frames in HEK293 cells when treated with 20uM Sal003, for 5 hours, n=6-12. (b) Expression of NLuc reporters with varying start codon mutations in HEK293T cells relative to the negative control GGG-NLuc, n=9. (c-d) Response of the AUG-NLuc and near cognate-NLuc reporters, co-transfected with the internal FLuc control, in HEK293T cells (c) co-transfected with either WT or S51D (phosphomimetic) eIF2α, n=12-15, or treated with (d) 2.5 μg/ml tunicamycin, n=9, or (e) 20 μM sodium arsenite, n=9, for 5 hours. Graphs represent mean ± SEM. Two-tailed Student's t test with Welch's correction, $**p < 0.01$; $***p < 0.001$, $****p<0.0001$.

## Supplementary Figure 6

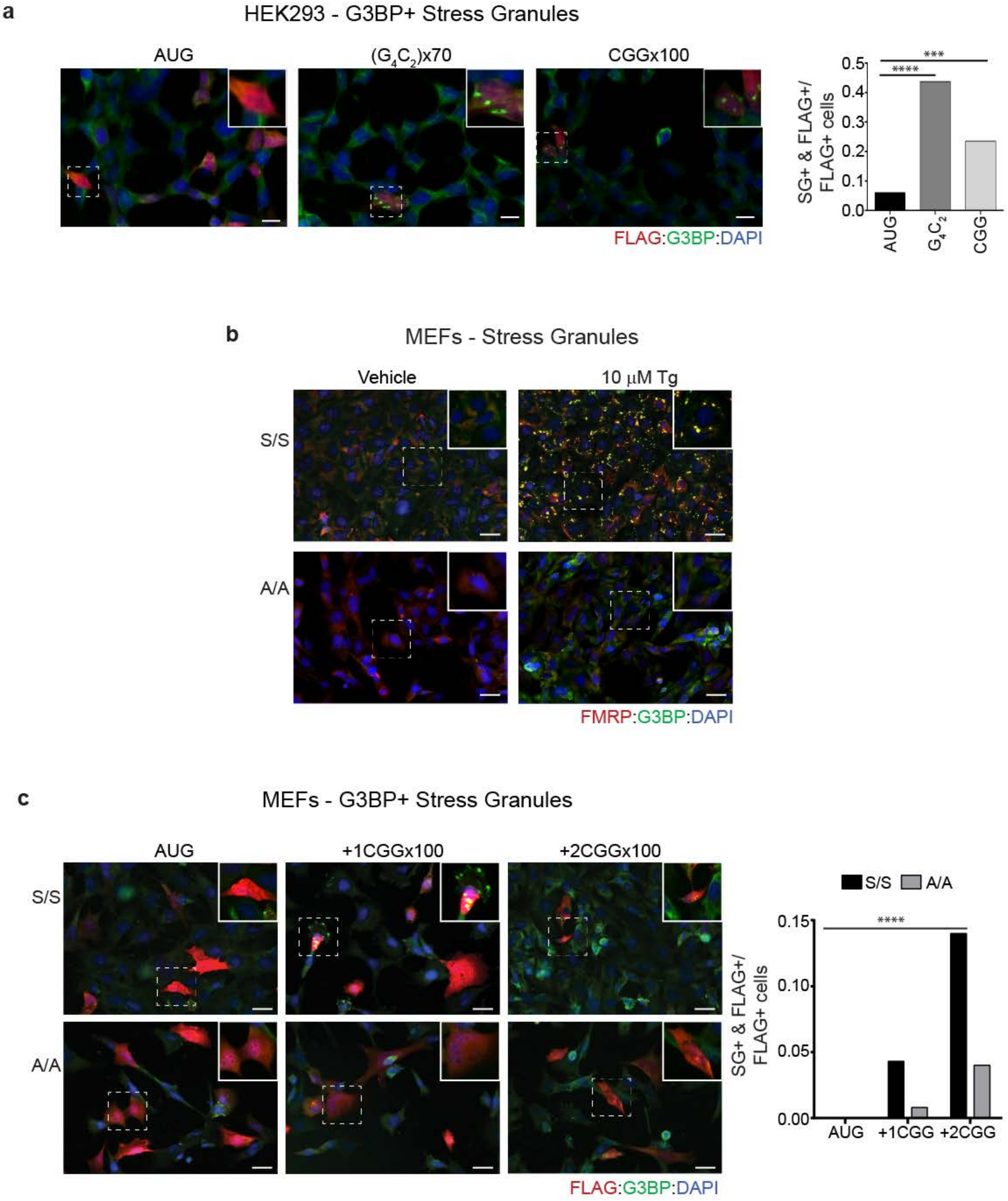

**Supplementary Figure 6: CGG and $G_4C_2$ repeats induce G3BP-positve stress granules in a phosphorylated-eIF2α dependent manner.**

(a) *Left*: Immunofluorescent images of HEK293 cells expressing control, $(G_4C_2)$x70, or CGGx100 reporters, scale bar=100 μm. *Right:* Quantification of the proportion of FLAG-positive cells with G3BP-positive stress granules (SGs) for each genotype, n>45. (b) Immunofluorescent images of WT and eIF2α- S51A/A MEFs treated with vehicle or 10 μM TG for 3 hours, scale bar=100 μm. (c) *Left:* Immunofluorescent images of WT and eIF2α-S51A/A MEFs expressing control, +1CGGx100, or +2CGGx100 RAN reporters, scale bar=100 μm. *Right:* Quantification of the proportion of FLAG-positive cells with G3BP-positive SGs for each genotype, n>40. FLAG marks reporter expressing cells, G3BP mark SGs. Fisher's exact test, ***$p < 0.001$.; ****$p<0.0001$.

# Supplementary Figure 7

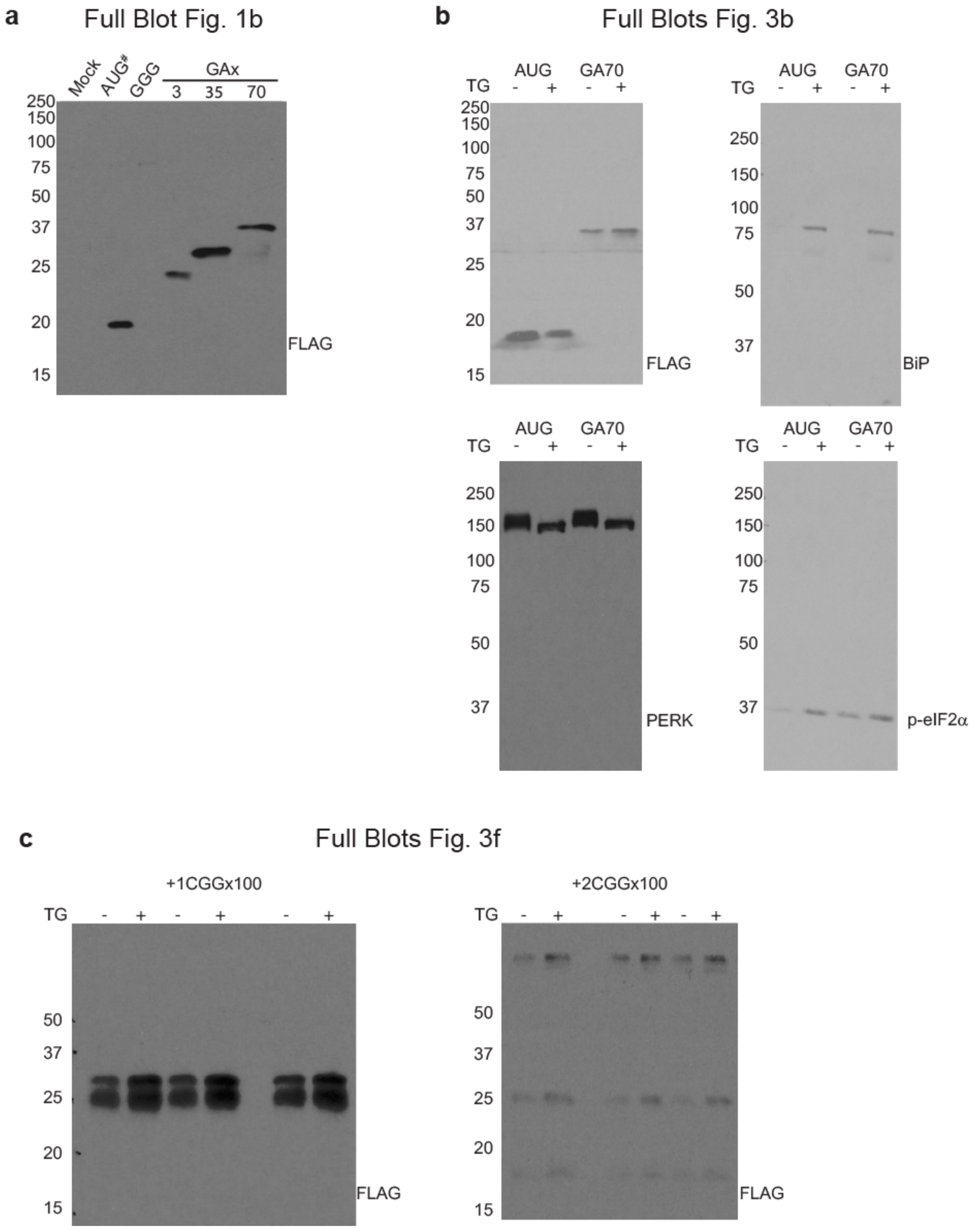


**Supplementary Figure 7: Full-length western blots of key experimental findings**

From (a) Fig. 1b (b) Fig. 3b and (c) Fig. 3f.

**Supplementary Table 1: Primers for reporter generation**

| Reporter | Forward Primer Sequence | Reverse Primer Sequence |
|---|---|---|
| PSP-GGG-NL-3xFLAG | CCGGTCTCGAGGTCCTCTTCCAGGGACCCA | CCGGTGGGTCCCTGGAAGAGGACCTCGAGA |
| GA frame PSP site | CCAGGGACCCGATGGGGTCTTCAC | AAGAGGACCTCGAGACCG |
| GP frame PSP site | CCTCGAGGTCCTCTTCCAGGGACCCGATGG | ACCGGTGGGCGCGCCCGG |
| GR frame PSP site | CCCTCGAGGTCCTCTTCCAGGACCCGATGG | |
| Intron1 CTG-CCC | GTAGCAAGCTCCCGAACTCAGGAGTCGC | AGGCTGCGGTTGTTTCCC |
| Intron1 AGG-AAA | TCTGGAACCAAAAGTCGCGCGC | GCTTGCTACAGGCTGCGG |
| Intron1 CTG-CCC and AGG-AAA | CTAAAAGTCGCGCGCTAGCGGCC | TTCGGGAGCTTGCTACAGGCTGCGTTG |
| Intron CTG-ATG | GTAGCAAGCTATGGAACTCAGG | AGGCTGCGGTTGTTTCCC |
| Intron1 AUG | ATGAGTCGCGCGCTATCTA | CCTGAGTTCCAGAGCTTG |
| AUG-GA | CTAGCTAACTAACACCATGGC | GGCCGCCATGGTGTTAGTTAG |
| AUG-GP | CTAGCTAACTAACACCATGGGGC | GGCCGCCCCATGGTGTTAGTTAG |
| AUG-GR | CTAGCTAACTAACACCATGGGC | GGCCGCCCATGGTGTTAGTTAG |
| CUG-NLuc | ACCCTGGTCTTCACACTCGAAGATTTC | GGCTTATTTACCAACAGTACCGGATTG |
| GUG-NLuc | ACCGTGGTCTTCACACTCGAAGATTTC | |
| ACG-NLuc | ACCACGGTCTTCACACTCGAAGATTTC | |

## Supplementary Table 2: C9RAN construct sequences

| Construct | Sequence |
|---|---|
| Nhe1-Intron1-GA70-PSP-GGG-NLuc-3xFLAG-PspOMI | GCTAGCGTGTGTGTTTTTGTTTTTCCCACCCTCTCTCCCCACTACTTGCTCTCACAGTACTCG<br>CTGAGGGTGAACAAGAAAAGACCTGATAAAGATTAACCAGAAGAAAACAAGGAGGGAAACAA<br>CCGCAGCCTGTAGCAAGCTCTGGAACTCAGGAGTCGCGCGCTAGCGGCCGGGGCCGGGGC<br>CGGGGCCGGGGCCGGGGCCGGGGCCGGGGCCGGGGCCGGGGCCGGGGCCGGGGCCGG<br>GGCCGGGGCAGGGGCCGGGGCCGGGGCCGGGGCCGGGGCCGGGGCCGGGGCCGGGGC<br>CGGGGCCGGGGCCGGGGCCGGGGCCGGGGCCGGGGCCGGGGCCGGGGCCGGGGCCGG<br>GGCCGGGGCCGGGGCCGGGGCCGGGGCCGGGGCCGGGGCCGGGGCCGGGGCCGGGGC<br>CGGGGCCGGGGCCGGGGCCGGGGCCGGGGCCGGGGCCGGGGCCGGGGCCGGGGCCGG<br>GGCCGGGGCCGGGGCCGGGGCCGGGGCCGGGGCCGGGGCCGGGGCCGGGGCCGGGGC<br>CGGGGCCGGGGCCGGGGCCGGGGCCGGGGCCGGGGCCGGGGCCGGGGCCGGGGCCGG<br>GGCCGGGGCCGGTCGTGGAAGGGTGGGCGCGCCcACCGGTCTCGAGGTCCTCTTCCAGGG<br>ACCCGATGGGGTCTTCACACTCGAAGATTTCGTTGGGGACTGGCGACAGACAGCCGGCTAC<br>AACCTGGACCAAGTCCTTGAACAGGGAGGTGTGTCCAGTTTGTTTCAGAATCTCGGGGTGTC<br>CGTAACTCCGATCCAAAGGATTGTCCTGAGCGGTGAAAATGGGCTGAAGATCGACATCCATG<br>TCATCATCCCGTATGAAGGTCTGAGCGGCGACCAAATGGGCCAGATCGAAAAAATTTTTAAG<br>GTGGTGTACCCTGTGGATGATCATCACTTTAAGGTGATCCTGCACTATGGCACACTGGTAATC<br>GACGGGGTTACGCCGAACATGATCGACTATTTCGGACGGCCcTATGAAGGCATCGCCGTGTT<br>CGACGGCAAAAAGATCACTGTAACAGGGACCCTGTGGAACGGCAACAAAATTATCGACGAGC<br>GCCTGATCAACCCCGACGGCTCCCTGCTGTTCCGAGTAACCATCAACGGAGTGACCGGCTG<br>GCGGCTGTGCGAACGCATTCTGGCGGACTACAAAGACCATGACGGTGATTATAAAGATCATG<br>ACATCGATTACAAGGATGACGATGACAAGTAAGGCCGCGACTCGAGAGGGCCC |
| Nhe1-Intron1-GP70-PSP-GGG-NLuc-3xFLAG-PspOMI | GCTAGCGTGTGTGTTTTTGTTTTTCCCACCCTCTCTCCCCACTACTTGCTCTCACAGTACTCG<br>CTGAGGGTGAACAAGAAAAGACCTGATAAAGATTAACCAGAAGAAAACAAGGAGGGAAACAA<br>CCGCAGCCTGTAGCAAGCTCTGGAACTCAGGAGTCGCGCGCTAGCGGCCGGGGCCGGGGC<br>CGGGGCCGGGGCCGGGGCCGGGGCCGGGGCCGGGGCCGGGGCCGGGGCCGGGGCCGG<br>GGCCGGGGCAGGGGCCGGGGCCGGGGCCGGGGCCGGGGCCGGGGCCGGGGCCGGGGC<br>CGGGGCCGGGGCCGGGGCCGGGGCCGGGGCCGGGGCCGGGGCCGGGGCCGGGGCCGG<br>GGCCGGGGCCGGGGCCGGGGCCGGGGCCGGGGCCGGGGCCGGGGCCGGGGCCGGGGC<br>CGGGGCCGGGGCCGGGGCCGGGGCCGGGGCCGGGGCCGGGGCCGGGGCCGGGGCCGG<br>GGCCGGGGCCGGGGCCGGGGCCGGGGCCGGGGCCGGGGCCGGGGCCGGGGCCGGGGC<br>CGGGGCCGGGGCCGGGGCCGGGGCCGGGGCCGGGGCCGGGGCCGGGGCCGGGGCCGG<br>GGCCGGGGCCGGTCGTGGAAGGGTGGGCGCGCCcACCGGTcCTCGAGGTCCTCTTCCAGG<br>GACCCGATGGGGTCTTCACACTCGAAGATTTCGTTGGGGACTGGCGACAGACAGCCGGCTA |

| | |
|---|---|
| | CAACCTGGACCAAGTCCTTGAACAGGGAGGTGTGTCCAGTTTGTTTCAGAATCTCGGGGTGT<br>CCGTAACTCCGATCCAAAGGATTGTCCTGAGCGGTGAAAATGGGCTGAAGATCGACATCCAT<br>GTCATCATCCCGTATGAAGGTCTGAGCGGCGACCAAATGGGCCAGATCGAAAAAATTTTTAA<br>GGTGGTGTACCCTGTGGATGATCATCACTTTAAGGTGATCCTGCACTATGGCACACTGGTAAT<br>CGACGGGGTTACGCCGAACATGATCGACTATTTCGGACGGCCcTATGAAGGCATCGCCGTGT<br>TCGACGGCAAAAAGATCACTGTAACAGGGACCCTGTGGAACGGCAACAAAATTATCGACGAG<br>CGCCTGATCAACCCCGACGGCTCCCTGCTGTTCCGAGTAACCATCAACGGAGTGACCGGCT<br>GGCGGCTGTGCGAACGCATTCTGGCGGACTACAAAGACCATGACGGTGATTATAAAGATCAT<br>GACATCGATTACAAGGATGACGATGACAAGTAAGGCCGCGACTCGAGAGGGCCC |
| Nhe1-Intron1-GR70-PSP-GGG-NLuc-3xFLAG-PspOMI | GCTAGCGTGTGTGTTTTTGTTTTTCCCACCCTCTCTCCCCACTACTTGCTCTCACAGTACTCG<br>CTGAGGGTGAACAAGAAAAGACCTGATAAAGATTAACCAGAAGAAAACAAGGAGGGAAACAA<br>CCGCAGCCTGTAGCAAGCTCTGGAACTCAGGAGTCGCGCGCTAGCGGCCGGGGCCGGGGC<br>CGGGGCCGGGGCCGGGGCCGGGGCCGGGGCCGGGGCCGGGGCCGGGGCCGGGGCCGG<br>GGCCGGGGCAGGGGCCGGGGCCGGGGCCGGGGCCGGGGCCGGGGCCGGGGCCGGGGC<br>CGGGGCCGGGGCCGGGGCCGGGGCCGGGGCCGGGGCCGGGGCCGGGGCCGGGGCCGG<br>GGCCGGGGCCGGGGCCGGGGCCGGGGCCGGGGCCGGGGCCGGGGCCGGGGCCGGGGC<br>CGGGGCCGGGGCCGGGGCCGGGGCCGGGGCCGGGGCCGGGGCCGGGGCCGGGGCCGG<br>GGCCGGGGCCGGGGCCGGGGCCGGGGCCGGGGCCGGGGCCGGGGCCGGGGCCGGGGC<br>CGGGGCCGGGGCCGGGGCCGGGGCCGGGGCCGGGGCCGGGGCCGGGGCCGGGGCCGG<br>GGCCGGGGCCGGTCGTGGAAGGGTGGGCGCGCCcACCGGTccCTCGAGGTCCTCTTCCAGG<br>GACCCGATGGGGTCTTCACACTCGAAGATTTCGTTGGGGACTGGCGACAGACAGCCGGCTA<br>CAACCTGGACCAAGTCCTTGAACAGGGAGGTGTGTCCAGTTTGTTTCAGAATCTCGGGGTGT<br>CCGTAACTCCGATCCAAAGGATTGTCCTGAGCGGTGAAAATGGGCTGAAGATCGACATCCAT<br>GTCATCATCCCGTATGAAGGTCTGAGCGGCGACCAAATGGGCCAGATCGAAAAAATTTTTAA<br>GGTGGTGTACCCTGTGGATGATCATCACTTTAAGGTGATCCTGCACTATGGCACACTGGTAAT<br>CGACGGGGTTACGCCGAACATGATCGACTATTTCGGACGGCCcTATGAAGGCATCGCCGTGT<br>TCGACGGCAAAAAGATCACTGTAACAGGGACCCTGTGGAACGGCAACAAAATTATCGACGAG<br>CGCCTGATCAACCCCGACGGCTCCCTGCTGTTCCGAGTAACCATCAACGGAGTGACCGGCT<br>GGCGGCTGTGCGAACGCATTCTGGCGGACTACAAAGACCATGACGGTGATTATAAAGATCAT<br>GACATCGATTACAAGGATGACGATGACAAGTAAGGCCGCGACTCGAGAGGGCCC |
